# Impacts of drainage beds covered with sand and wood shavings on the comfort behaviour of dairy buffalo in a temperate climate

**DOI:** 10.1186/s13620-020-0157-1

**Published:** 2020-03-02

**Authors:** Lin Li, Ping Liu, Lulu Guo, Fulan Zhang, Jinhui Pu, Huaming Mao, Zhaobing Gu

**Affiliations:** Faculty of Animal Science and Technology, Yunnan Agricultural University, Kunming, 650201 China

**Keywords:** Buffaloes, Behaviour, Bedding materials, Drainage beds

## Abstract

**Background:**

Comfortable beds play an important role in increasing the ruminant and milk production efficiency of dairy buffalo. In loose housing systems, cow lying comfort depends on both the bedding materials and bed base.

**Results:**

Buffaloes spent more lying time on sand beds at depth of 15 and 20 cm (S-15 and S-20) than on beds of 10 cm (S-10a) beds or in the feed alley in Exp1 (*P* < 0.01). No significant difference in the cow comfort index (CCI) was detected between S-10a and S-15; however, both showed higher CCI than that of the S-20 bed. In Exp2, buffaloes spent more time lying on the wood shavings at depth of 15 cm (WS-15) bed followed by the WS-20 and S-10b beds, respectively (*P* < 0.01), and CCI was greater in the WS-15 bed than in the S-10b and WS-20 beds (*P* < 0.05).

**Conclusion:**

A depth of sand or wood shavings at 15 cm can meet the lying comfort requirements of dairy buffaloes when bedding materials are used above drainage beds.

## Background

There are 199 million buffaloes in the world, with 194 million buffaloes distributed in Asia (http://faostat3.fao.org/compare/Q/QA/E). India, China and Pakistan contain most of the world’s buffalo population. India produces 70% of the world’s buffalo milk, and buffalo milk from Pakistan, China and Egypt make up 20, 5 and 4% of the world’s production, respectively [1]. River buffaloes and crossbred buffaloes (Swamp × River) are ruminant animals that have important roles in milk production. High feed efficiency and milk performance are closely associated with more ruminant behaviour, which is positively affected by lying comfort and the total time spent lying. Ideally, dairy cattle need to spend 12–14 h lying [2].

Bedding materials affect the production and lying comfort of dairy cattle [3]. Concrete is widely used as the flooring material in dairy farms as it is convenient to manage; however, dairy cattle are reluctant to use it as a lying area due to its uncomfortable surface. Lying at the feed alley for a long time may cause injury to teats and mastitis in dairy cattle. Comfortable bedding materials increase lying times and the flow of blood to the udder, which is conducive to increasing milk yield [2]. Mattresses with cushions are also commonly used as bedding material to increase milk yield [4, 5]. However, mattresses that are not drainable are inappropriate as bed bases, when dairy cattle are loose housed without the restriction of stalls.

Sand is one type of inorganic bedding material that is soft and cool and facilitates urine evaporation, which is beneficial for reducing hock disease [6, 7], mastitis morbidity and the incidence of lameness [8, 9]. Straw, sawdust and wood shavings are also available as bedding material for dairy animals; however, the bed base also plays an important role in lying comfort. The bedding surfaces of free-stalls are scarcely soiled by faeces and urine, but free-stalls inhibit the expression of normal behaviours. Loose housing systems meet this requirement, but the bedding surfaces become soiled with faeces and urine. The disadvantages of loose housing systems around keeping bedding materials dry and clean can be avoided with the use of slatted floors; however, this endangers cattle claw health and locomotion [10].

A dairy cow produces approximately 30 kg of faeces and 15 kg of urine per day [11]. At the same intake of dry matter, buffalo defecate a higher amount of faeces than cattle [12]. Manure accumulation on bedding surfaces causes risks of lameness, heel erosion and mastitis for cows [13]. Many factors affect the defecation behaviour of cows. Loose housed dairy cattle in timed feeding show a higher frequency of defecation than that of cows provided food ad libitum [14]. Dry dairy cattle performed 42% of their defecations from 20:00 to 08:00 h [15]. When given high amounts of concentrate, dairy cattle showed high numbers of defecations [11]. However, information about the effects of defecation behaviours and faeces distribution on the hygiene of bedding material for buffaloes is scarce.

A drainage bed was constructed to keep bedding materials dry and clean. Drainage bed bases covered to a certain depth with sand or wood shavings may provide different levels of lying comfort for buffaloes. We also investigated the defecation behaviour and faeces distribution of buffaloes to provide guidance for bedding material management.

## Materials and methods

### Drainage bed system construction

**Fig. 1:**
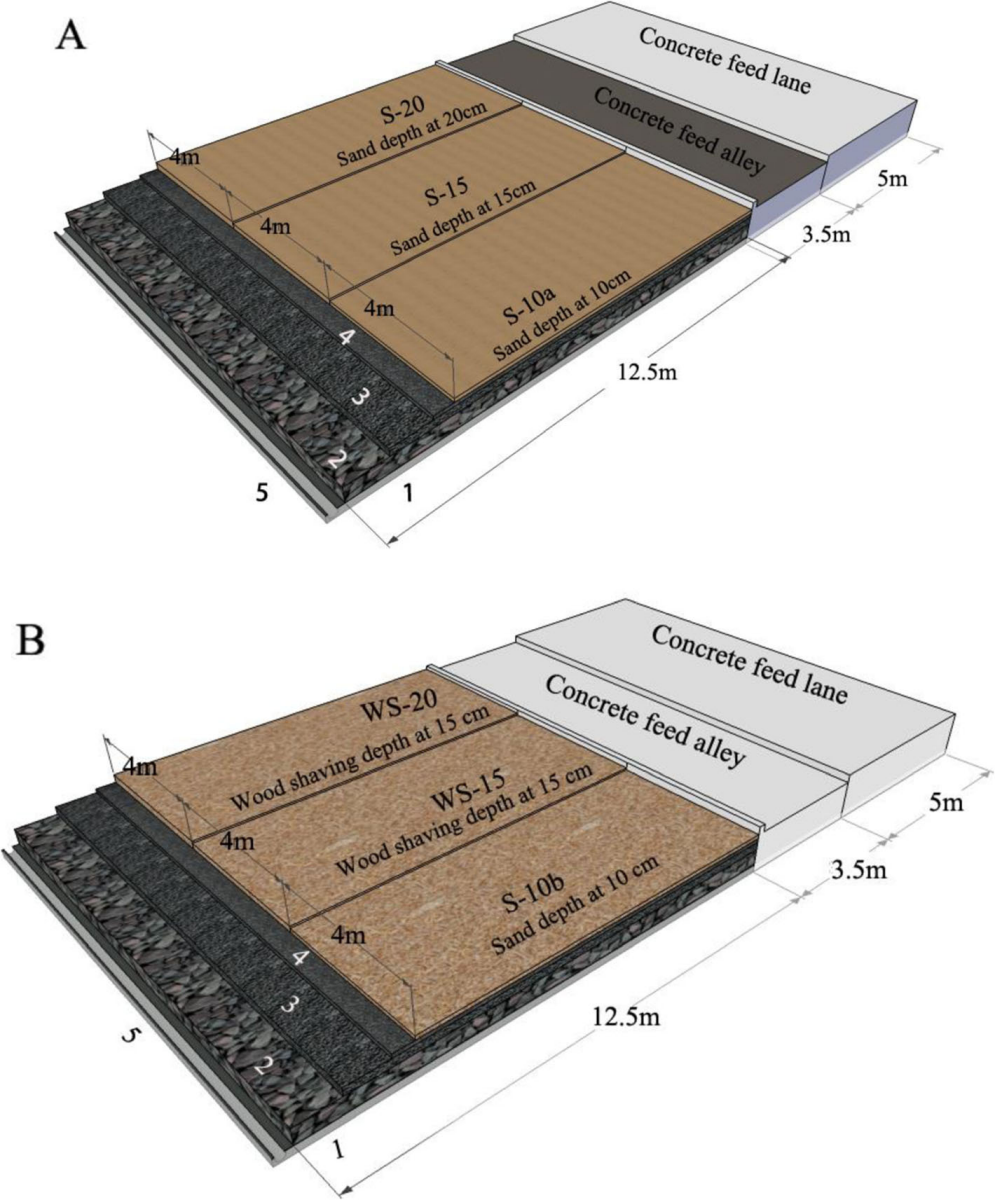
Pen dimensions, and drainage beds covered with sand and/ or wood shaving at different depth in Exp1 (**a**) and Exp2 (**b**). 1- impervious concrete layer, 2- rubble stone layer at depth of 30 cm, 3- cobblestone layer at depth of 20 cm, 4- gravel layer at depth of 10 cm, 5- gutter

The length and width of the bed bases were 12.5 m and 12.0 m, respectively. The bed base included three parts: (1) The bottom layer that was made from impervious concrete with a 2% slope; (2) The middle layer that was made from rubble stone with a depth of 30 cm; and (3) The top layer that was made from 20 cm deep cobblestone, followed by 10 cm of gravel. The bed base was divided into three cubicles (12.5 m × 4.0 m) with round-timber and the buffaloes had access to all three cubicles in Exp1. The three cubicles were covered with sand at a depth of 10 cm (S-10a), 15 cm (S-15) and 20 cm (S-20) (Fig. 1a). River sand without silt, sieved over a 2-mm screen, was used in this experiment. In Exp2, another bed base with the same dimensions was covered with a 10 cm thick layer of sand (S-10b) or 15 cm (WS-15) and 20 cm (WS-20) of wood shavings (Fig. 1b). The length of the wood shavings was 20 mm to 40 mm. The bedding materials were manually groomed and supplied at timely intervals to maintain the same depth during the two field trials.

### Animal management

The study was conducted in Dehong, China in December 2018. Fifteen healthy buffaloes with similar body conditions were used in the study and each bedding area (50 m^2^) met the requirements for lying areas for buffaloes. Buffaloes were fed a whole corn silage and concentrate, and they had free access to fresh drinking water.

The cow comfort index (CCI) was used to evaluate the lying comfort of each bed covered with the different bedding materials to certain depths. We recorded the number of buffaloes lying on a bedding area at 10:00, 16:00 and 20:00 h to calculate CCI. CCI is calculated by dividing the number of buffaloes lying on a bedding surface by the total number of buffaloes [16, 17]. Faeces in the feed alley and on the different bedding surfaces were collected for weighing.

### Animal behavioural observation

Fifteen dairy buffaloes were observed using continuous sampling for 96 h. Buffalo behaviours including standing, lying and defecation were measured. A buffalo was considered to be lying when her body trunk was in contact with the ground. Buffaloes were considered standing when their body weight was supported by four legs. Defecation was defined as occurring when faeces was observed dropping down from the anus. After finishing the field trail, animal behavioural data were extracted from video files to evaluate the preferences of buffalo for bedding surfaces.

### Statistical analysis

Analyses were performed using IBM SPSS 21.0 (IBM Corporation, New York, N.Y., USA). Behavioural parameters (lying, standing and defecation at each location) were obtained from individual buffaloes. Behavioural data and the amount of faeces were analysed using one-way analysis of variance.

## Results

The total time budgets of the dairy buffaloes in Exp1 are summarised in Table 1. Buffaloes spent more time standing (including walking and feeding) in the feed alley than standing in the S-10a, S-15 and S-20 beds (*P* < 0.01). In terms of standing without feeding, buffaloes spent less time in the feed alley and the S-10a bed than in the S-15 and S-20 beds, and no significant difference was detected between the S-15 and S-20 beds (*P >* 0.05). No buffalo selected the feed alley as a lying area. The most preferred bedding surface was S-15, followed by S-20. Buffaloes spent the least time lying in the S-10a bed. No differences in the time budgets for defecation was detected between the feed alley and the three bedding surfaces (*P* < 0.05).

**Table 1:** Time allocation (min) in the feed alley and drainage bed covered with sand at different depths (means ± SE) in Exp1

Location	All standing	Standing only	Lying	Defecation
Alley	239.0 ± 17.9^A^	103.0 ± 14.5^A^	–	0.5 ± 0.1
S-10a	105.9 ± 9.0^B^	105.9 ± 9.0^A^	93.3 ± 27.7^A^	0.4 ± 0.0
S-15	180.3 ± 18.3^C^	180.3 ± 18.3^B^	408.0 ± 39.6^B^	0.6 ± 0.1
S-20	172.3 ± 13.1^C^	172.3 ± 13.1^B^	241.3 ± 43.7^C^	0.6 ± 0.1

^A, B, C^ Means within variable having different superscript letters differ (*P* < 0.01)

Dairy buffalo behaviours in Exp2 are shown in Table 2. No significant difference was detected in standing time between the feed alley and the WS-15 bed (*P >* 0.05); however, standing time was significantly higher than that for the S-10 b and WS-15 beds (*P* < 0.01). After subtracting the feeding time, buffaloes spent less standing time in the feed alley than that of the three bedding surfaces. No buffalo selected the feed alley as a lying area in Exp2 and time spent lying was significantly different between the S-10b, WS-15 and WS-20 beds (*P* < 0.01). Buffaloes preferred to lie on the WS-15 bed rather than the WS-20 and S-10b beds. Defecation time in the feed alley was lower than that of the S-10b, WS-15 and WS-20 beds.

**Table 2:** Time allocation (min) in the feed alley and drainage bed with sand or wood shavings at different depths (means ± SE) in Exp2

Location	All standing	Standing only	Lying	Defecation
Alley	209.7 ± 9.6^A^	72.0 ± 9.0^A^	–	0.4 ± 0.1^A^
S-10b	120.4 ± 14.6^aB^	120.4 ± 14.6^aB^	146.8 ± 26.4^A^	0.5 ± 0.0^AB^
WS-15	211.8 ± 13.5^A^	211.8 ± 13.5^C^	343.5 ± 28.9^B^	0.8 ± 0.1^BC^
WS-20	155.9 ± 12.8^bB^	155.9 ± 12.8^bB^	251.9 ± 30.7^C^	0.6 ± 0. 1^BC^

^a,b^ Means within variable having different superscript letters differ (*P* < 0.05); ^A, B, C^ Means within variable having different superscript letters differ (*P* < 0.01)

The CCI for dairy buffaloes is summarised in Table 3. In Exp1, when buffaloes were loose housed with well-drained beds covered with different depths of sand, S-10a and S-15 showed higher CCI than that of S-20 (*P* < 0.05). In Exp2, buffaloes in the WS-15 bed had a higher CCI than those in the S-10b and WS-20 beds; however, no significant difference was detected between the S-10b and WS-20 beds (*P >* 0.05).

**Table 3:** Cow comfort index (CCI) on drainage beds covered with sand or wood shavings at different depths (means ± SE)

Exp1	CCI	Exp2	CCI
S-10a	27.22 ± 5.77^a^	S-10b	17.78 ± 4.44^a^
S-15	26.67 ± 4 .92^a^	WS-15	23.70 ± 6.60^b^
S-20	17.22 ± 4.38^b^	WS-20	15.56 ± 4.00^a^

^a,b^ Means within variable having different superscript letters differ (*P* < 0.05)

The amount of faeces at different locations is shown in Table 4. The daily amount of faeces was significantly greater in the feed alley than that in the S-10a, S-15 and S-20 beds in Exp1. Buffalo faeces in the alley was also greater than that in the S-10b, WS-15 and WS-20 beds (*P* < 0.01); however, no significant differences were detected among the three bed in Exp2 (*P* > 0.05).

**Table 4:** Amount of faeces (kg) of dairy buffaloes at different defecation locations (means ± SE)

Defecation location	Amount of faeces	Defecation location	Amount of faeces
Alley	143.8 ± 17.6^A^	Alley	187.1 ± 7.3^A^
S-10a	55.8 ± 13.9^B^	S-10b	43.7 ± 8.3^B^
S-15	25.1 ± 5.5^B^	WS-15	35.2 ± 3.7^B^
S-20	35.4 ± 4.0^B^	WS-20	25.4 ± 3.8^B^

^A, B^ Means within variable having different superscript letters differ (*P* < 0.01)

## Discussion

Lying is a high priority for dairy cattle [18] and bedding types affect their lying behaviours. Dairy cattle prefer straw to sand [19] and the time spent lying has been found to be greater on sand and sawdust than on a mattress [20]. The least favourite flooring was concrete, causing foot erosion and lameness. No buffalo selected the concrete feed alley as a lying area in the present study, which is consistent with a previous study that showed that dairy cattle decreased their lying time on wet and muddy floors [21]. Due to its high cost, straw is rarely used as a bedding material in the production of buffaloes of buffaloes. Owing to their low cost, sand and wood shavings were selected as bedding materials in the present study.

Dairy buffaloes showed a preference for lying in S-15 beds rather than S-20 beds in Exp1, which is contrary to a previous study that showed lying time decreased on a thin layer of sand bed [22]. Dry sand at a constant depth of 15 cm above the bed may be enough to maintain lying comfort for dairy buffaloes. Another reason may be that sand at a depth of 20 cm is too soft when on top of a drainage bed, affecting posture during the transition from standing to lying. After subtracting the feeding time, time spent standing in the feed alley was lower than that of the three bedding surfaces, indicating that buffaloes are reluctant to stand on the hard concrete feed alley floors unless they are feeding.

Consistent with the results in Exp1, buffaloes also had a higher preference for lying on WS-15 rather than WS-20 beds (*P* < 0.01), with the least favourite bed being the S-10b bed (10 cm depth; Exp2). Wood shavings may provide greater lying comfort in temperate climates and support when transitioning between standing and lying. Another possible explanation is that the WS-15 beds could provide a comfortable lying environment when the depth of the WS is manually groomed and replenished in a timely manner to keep it at a similar depth above the drainage bed. The key characteristic of this bed base is that it keeps bedding materials dry and clean by draining away urine, which may increase the lying time for buffaloes.

The CCI was inconsistent with the time budget for lying in Exp1. Cook et al. [16] also failed to find an association between CCI and total lying time. Time spent lying may be a reasonably good evaluation of the lying comfort. Faeces were mainly distributed in the feed alley in Exp1 and Exp2. From the characteristic posture observed, such as raised tail, spread hind feet, and arched back, during defecations [11], cattle may have made conscious attempts to avoid contamination with urine. Dairy cattle defecated mainly in standing postures, especially in the feed alley [23]. Time spent standing by buffaloes was greater in the feed alley than on the other bedding surfaces in the present study, which may explain the high amount of faeces in this area. Robichaud et al. (2011) found only a small portion of cattle defecated in a lying posture [23] and no buffalo in our loose housing systems did this in a lying posture in our two experiments.

Defecation time in the feed alley was lower than that in the S-10b, WS-15 and WS-20 beds, and most of the buffalo faeces were distributed in the feed alley. As there are no special feed alleys in many small-scale and family farms, buffalo body surfaces are often very dirty, which affects milk quality and their welfare. Based on the distribution of faeces, it is necessary to design a special feed alley to decrease the amount of faeces above the bedding material. Based on this evaluation of the defecation behaviour and faeces distribution, faeces were collected twice daily, making bedding surfaces dry and clean. We therefore did not evaluate buffalo cleanliness. Wood shavings, as a type of organic bedding material, may cultivate fly larvae and coliforms, as occurs in long straw, resulting in mastitis outbreaks [24, 25]. Future research should evaluate the hygienic safety of wood shavings on drainage beds in hot summers.

## Conclusions

The results from the present study indicated that sand at a depth of 15 cm on drainage beds can meet the requirements for lying comfort of dairy buffaloes. When using wood shavings as bedding material on a drainage bed, dairy buffaloes preferred to lie on it at a depth of 15 cm. A special feed alley design for feed and defecation can keep bedding material dry and clean to increase lying comfort.

## Data Availability

All data generated or analyzed during this study are included in this published article.

## Abbreviations

CCI: Cow comfort index
S-10a: Sand beds at depth of 10 cm in Exp1
S-15: Sand beds at depth of 15 cm
S-20: Sand beds at depth of 20 cm
WS: Wood shavings
WS-15: Wood shavings at depth of 15 cm
WS-20: Wood shavings at depth of 20 cm
